# Verification and validation of bioinformatics software without a gold standard: a case study of BWA and Bowtie

**DOI:** 10.1186/1471-2105-15-S16-S15

**Published:** 2014-12-08

**Authors:** Eleni Giannoulatou, Shin-Ho Park, David T Humphreys, Joshua WK Ho

**Affiliations:** 1 Victor Chang Cardiac Research Institute, Darlinghurst, NSW, Australia; 2 The University of New South Wales, NSW, Australia

**Keywords:** bioinformatics software testing, software quality assurance, metamorphic testing, genomic medicine

## Abstract

**Background:**

Bioinformatics software quality assurance is essential in genomic medicine. Systematic verification and validation of bioinformatics software is difficult because it is often not possible to obtain a realistic "gold standard" for systematic evaluation. Here we apply a technique that originates from the software testing literature, namely *Metamorphic Testing *(MT), to systematically test three widely used short-read sequence alignment programs.

**Results:**

MT alleviates the problems associated with the lack of gold standard by checking that the results from multiple executions of a program satisfy a set of expected or desirable properties that can be derived from the software specification or user expectations. We tested BWA, Bowtie and Bowtie2 using simulated data and one HapMap dataset. It is interesting to observe that multiple executions of the same aligner using slightly modified input FASTQ sequence file, such as after randomly re-ordering of the reads, may affect alignment results. Furthermore, we found that the list of variant calls can be affected unless strict quality control is applied during variant calling.

**Conclusion:**

Thorough testing of bioinformatics software is important in delivering clinical genomic medicine. This paper demonstrates a different framework to test a program that involves checking its properties, thus greatly expanding the number and repertoire of test cases we can apply in practice.

## Background

The advent of high-throughput Next Generation Sequencing (NGS) technologies has greatly accelerated the pace of disease gene discoveries and has revolutionised the diagnosis and management of human genetic diseases and cancer [1-5]. Being able to reconstruct the genetic make-up of an individual and accurately predict the effect of pathogenic genetic variants is essential for genetic counselling and making informed decisions regarding medical treatment. The age of personalised genomic medicine is upon us. New bioinformatics tools are being developed at a very rapid pace to analyse such datasets and to cope with the constant generation of new types of "omic" data [6].

Software quality assurance becomes especially critical if bioinformatics tools are to be used in a translational medical setting, such as analysis and interpretation of Whole Exome Sequencing (WES) or Whole Genome Sequencing (WGS) data. We must ensure that only validated algorithms are used, and that they are implemented correctly in the analysis pipeline. More importantly, the computed results must satisfy the general expectation of their intended users. Recently it has been shown that the concordance of multiple widely used variant-calling pipelines is very low (across 15 exomes, as low as 57.4% for single nucleotide variant calling, and 26.8% for indel calling) [7]. A similarly disturbing level of disagreement is also observed when using different variant annotation programs to annotate genetic variants even when the same transcript definition is used [8]. Considering there is only one ground truth, the high level of discrepancy is troubling, and is telling us that even the most popular bioinformatics tools to date can generate results with a non-negligible false positive or false negative rate. False positives and false negatives are both potentially a huge issue. Although false positives can easily be distinguished from true positives through external validation, such as Sanger sequencing, it is almost impossible to systematically distinguish false negatives from the vast number of true negatives.

Previous work on scientific software evaluation has shown that numerical disagreement between programs of scientific computation grows at around the rate of 1% in average absolute difference per 4000 lines of implemented code and that the nature of this disagreement is non-random [9]. Most recent scientific studies, especially in the area of bioinformatics and computational biology, deal with large and complex datasets and complicated algorithms. This complexity has made the replication of published findings difficult to pursue. In addition, not all users understand fully the intended usage and limitations of a scientific program [10]. Errors or limitations of the computer code utilised could go undetected with possible negative effects on future research [11]. Therefore, there has been an emerging issue of scientific peer-review of computer code in order to minimise errors or limitations that would prevent other researchers from replicating published findings [12]. Most importantly, there have been numerous published papers that attempt to train scientists to adopt best practices for scientific computing [13-17].

Software testing is defined as the process of actively identifying potential faults in a computer program [18,19]. This process can be used for two purposes: to ensure the program is correctly implemented against the specification (*i.e*., verification), and to ensure the correct specification is used against the desired user requirement (*i.e*., validation). Many software testing strategies have been developed, most of them are widely used in industry with varying effectiveness [20].

Performing testing systematically and automatically on bioinformatics programs is not as trivial as one would have imagined. It is often difficult, if not impossible, to define a gold standard mechanism to decide if the output of the target program is correct, given any possible input. This mechanism is referred to as an *oracle *in the software testing field. If a test oracle exists, we can apply a large number and variety of test cases to test a program since the correctness of the output can be verified using the oracle. Without a tangible oracle, the choice of test cases is greatly limited to those special test cases where the expected outputs are known or there exists a way to easily verify the correctness of the testing results. The problem is that the bioinformatics tools used in genomic medicine applications often lack an oracle, which greatly limits our ability to perform testing systematically and automatically. Currently most software developers test their programs using a small number of simulated test cases, or compare their programs with other existing programs that are expected to give the same results [21,22]. Both approaches are effectively an attempt to approximate a gold standard for testing. Nonetheless, there are clear shortcomings to both approaches. Simulation data may not truly reflect the characteristics of real data, and it is unclear what the best way is to determine ground truth if multiple programs give different results. In this manuscript, we demonstrate how we can systematically generate and check the correctness of many test cases without the need of a gold standard. Our strategy relies on a software testing technique called Metamorphic Testing (MT) [23-26]. This approach alleviates the oracle problem by using some problem domain-specific properties, namely metamorphic relations (MRs), to verify the testing outputs. The central idea is that although it is impossible to directly test the correctness of any given test case, it is easy to verify the relationships of the output generated by multiple executions of a program. In other words, MT tests for properties that users expect of a correct program.

We have previously utilised MT to test a range of bioinformatics and machine learning programs [27-29]. Our work represented a significant step towards software reliability in bioinformatics. In this work we aim to extend our contribution in this field by applying MT to three commonly used NGS short-read alignment programs that have been used widely by the international academic community: BWA [30], Bowtie [31] and Bowtie2 [32]. Our goal is not to present another comparison of short read alignment programs -- this has been previously studied extensively [33,34]. This paper is a case study on how we can adapt a state-of-the-art software testing technique (Metamorphic Testing) to systematically test several user expectations, which is useful for verifying and validating several widely used bioinformatics programs. We develop a fully automated software testing tool that can be used to systematically assess the expected behaviour of these programs. We also investigated the potential effect on variant calling if the alignment algorithms fail to satisfy some of the user expectations. This work serves as a case study for demonstrating the application of an intuitive testing framework for systematic verification and validation of bioinformatics software.

## Methods

### Metamorphic testing

**Figure 1:**
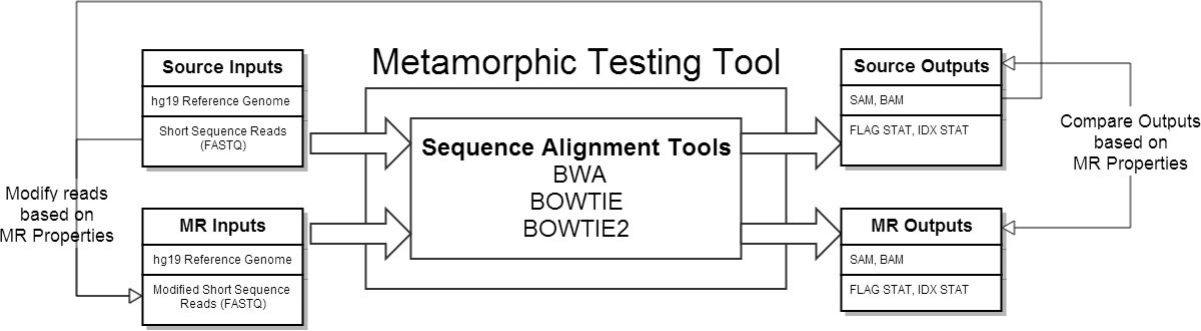
**Metamorphic Testing Tool**. Design of the Metamorphic Testing Tool applied to NGS short-read alignment software.

The design of our MT tool is shown in Figure 1. Short sequence reads (in FASTQ format) are aligned to the hg19 reference genome using three different sequence alignment tools: BWA (v0.7.5a) [30], Bowtie (v1.0) [31] and Bowtie2 (v2.1) [32]. The original "source" input, which can be based on simulated or real data, is modified by one or multiple MRs. Here we propose nine MRs based on expected properties of the software (*i.e*., what a regular user expects from this program). After the application of each MR on the input to generate a modified "follow-up" input, the program is executed again, and the output is tested against the expected relationship between the output of the source and follow-up input, as specified in the MRs. In other words, a MR serves two purposes: (1) generation of additional test cases by modifying the source input, and (2) checking the relationship between the outputs produced by the execution of the "source" and "follow-up" test cases. It should be noted that in general many follow-up test cases can be derived from a single source test case input based on one MR. In this study, we restrict our analysis to one source and one follow-up test case for each run of MT. The expected results vary according to each MR but can indicate whether failure is detected. Failures can imply various possibilities ranging from a deviation of the design of the software and user expectation (*i.e*., validation) to errors in the software implementation (*i.e*., verification).

The MT tool is implemented as a Unix shell script. The source code and documentation of the script is available at https://sourceforge.net/projects/mr-test/. Manipulation of the resulting output BAM files is performed with samtools (v0.1.19) [35].

#### Metamorphic relations

We designed the following set of MRs that aim to capture the expected behaviour of every short-read alignment software. It should be noted that the MRs were not designed based on the algorithm or implementation of a specific sequence aligner, but based on user expectations of the intended behaviour of a good sequence aligner. The description here is for a paired-end sequencing dataset, but it can be easily applied to single-end datasets too. We denote the reads of the two ends as Read1 and Read2.

**MR1: Random permutation of reads**. The reads in the FASTQ files are reshuffled. The permutation is the same for Read1 and Read2 reads. We expect the output mapping to be the same as the original output.

**MR2: Reverse complement of reads**. Both Read1 and Read2 are reverse complemented and their corresponding quality values are reversed to match the nucleotide bases. Their order in the algorithm input is reversed. We expect the output mapping to be the same.

**MR3: Addition of reads**. The input reads in the FASTQ files (both in Read1 and Read2 files) are duplicated. We expect the output of all original reads to map to the same locations with the additional reads mapping to the same locations as their duplicates.

**MR4: Removal of reads**. Half of the reads in the input FASTQ files (both in Read1 and Read2 files) are removed. We expect the output mapping of the non-removed reads to remain at the same locations. The resulting sequencing coverage should be half of the original mapping.

**MR5: Extension of reads**. After initial mapping, each read is extended by 20 bp to the 3' or 5' end of the read, with high quality score, based on the reference genome sequence. We expect the output mapping to remain the same.

**MR6: Unmapped reads**. After initial mapping, only the unmapped reads are selected and remapped against the reference genome. We expect that none of the reads will be mapped.

**MR7: Mapped reads**. After initial mapping, only the mapped reads are selected and remapped against the reference genome. We expect that all of the reads will be mapped. For paired-end reads only the properly paired reads are remapped.

**MR8: Quality score increase of reads**. After initial mapping, the quality score for all mapped sequences is increased. We expect the output mapping to remain the same.

**MR9: Correction of errors or mismatches in the mapped reads**. After initial mapping, the mapped reads are selected and any mismatch or error is corrected in the reads. We expect the output mapping to remain the same.

### Simulated data

Metamorphic testing properties can be applied to any sample, since there is no need for a gold standard. To demonstrate its utility on a range of input test cases, we apply MT on both simulated and real datasets. In terms of simulated data, we used simNGS and simLibrary (http://www.ebi.ac.uk/goldman-srv/simNGS/) to simulate NGS short-read datasets based on the human reference genome (hg19) of varying number of reads (10^3^, 10^4^, 10^5^, 10^6^, and 10^7^) in order to assess the effect of the total number of reads in our ability to detect limitations in the alignment software.

### HapMap data

One of the main advantages of MT is that we can test real data instead of only simulated data. We chose to apply MT to one widely studied HapMap sample that has been exome sequenced using Illumina HiSeq 2000 as part of the 1000 Genomes Project [36,37]. FASTA files with run ID SRR716647 (from study SRP004078) were downloaded from the 1000 Genomes FTP site (ftp://ftp.1000genomes.ebi.ac.uk/vol1/ftp/). The sequencing was paired-end with 26,892,758 Read1 and Read2 sequences. In addition, we downloaded a single-end dataset of 569,554 reads. This sequencing run was from HapMap individual NA12872, a male unaffected sample from 1000 Genomes CEPH (Utah residents with ancestry from Northern and Western Europe). Since the metamorphic properties require extensive manipulation of the FASTQ files, we chose this run as a representative example of a sequenced exome without having a very large file size that would limit our analysis.

### Variant calling using GATK

In order to assess the downstream effects of violating several key MRs (*e.g.*, MR1, MR5 and MR7) during sequence alignment on downstream genetic variant calling, we ran a commonly used variant calling pipeline on a HapMap dataset. After BWA, Genome Analysis Toolkit (GATK) (v2.6) was employed for variant calling [38]. Since our MRs do not apply any filtering on the BAM (mapping) files, the analysis was repeated after considering only the uniquely mapped reads.

## Results and discussion

### Limitations of short-read alignment software

We tested the performance of three widely used short-read alignment tools (BWA, Bowtie and Bowtie2) on simulated sequencing runs of varying coverage (10^3^, 10^4^, 10^5^, 10^6^, and 10^7 ^total number of reads in each FASTQ file). Table 1 shows the MR results for the three tools (BWA, Bowtie and Bowtie2) respectively. We additionally applied the same MRs to an exome sequencing run of HapMap sample NA12872 for both paired and single end datasets. Table 2 shows the results (indicating failure or pass) after applying 9 MRs for the paired and single end data respectively. Failure (F) indicates difference between the resulting alignments. This difference could be due to only a few or multiple reads mapping differently after the application of MT.

**Table 1:** Results of MT applied to three short-read alignment programs (BWA, Bowtie and Bowtie2) ran on the paired-end sequencing simulated reads of varying number.

MRs	10^3^	10^4^	10^5^	10^6^	10^7^
**BWA**					

MR1	F	F	F	F	F
MR2	F	F	F	F	F
MR3	F	F	F	F	F
MR4		F	F	F	F
MR5	F	F	F	F	F
MR6					
MR7				F	F
MR8					
MR9					

**Bowtie**					

MR1					
MR2	F	F	F	F	F
MR3					
MR4					
MR5	F	F	F	F	F
MR6					
MR7					
MR8	F	F	F	F	F
MR9					

**Bowtie2**					

MR1					
MR2	F	F	F	F	F
MR3					
MR4					
MR5	F	F	F	F	F
MR6					
MR7					
MR8	F	F	F	F	F
MR9					

F (Failure).

**Table 2:** Results of MT applied to 3 NGS short-read alignment programs ran on the paired-end and single end sequencing reads of HapMap sample NA12872.

MRs	BWA	BOWTIE	BOWTIE2
**Paired-end sequencing reads**			

MR1	F		
MR2	F	F	F
MR3	F		
MR4	F		
MR5	F	F	F
MR6			
MR7	F		
MR8		F	F
MR9	F		F

**Single-end sequencing reads**			

MR1	F		
MR2	F	F	F
MR3	F		
MR4			
MR5	F	F	F
MR6			
MR7			
MR8		F	F
MR9	F		F

F (Failure).

We found that the expected behaviour that is encoded by MR1 (Random permutation of reads) and MR3 (Addition of reads) does not hold for BWA for either simulated or real data (NA12872; single and paired-end) but passed our tests for both Bowtie and Bowtie2. Similarly, properties MR4 (Removal of reads) and MR7 (Mapped reads), although they hold for Bowtie and Bowtie2, they fail for BWA for simulated paired-end data that are above some sequencing coverage (*>*10^3 ^for MR4 and *>*10^7 ^for MR7). Metamorphic relations MR2 (Reverse complement of reads) and MR5 (Extension of reads) fail for all three algorithms and input data whereas on the other hand MR6 (Unmapped reads) is never violated. Finally, we found that MR8 (Quality score increase of reads) fails for Bowtie and Bowtie2 but not for BWA and MR9 (Correction of errors or mismatches in the mapped reads) fails for BWA and Bowtie2 only when applied on the real sequencing run (NA12872).

Following our "black-box" testing, we attempted to explain the above results by investigating the nature of failure. We concluded that MR2 (Reverse complement of reads) and MR5 (Extension of reads) although they could be desirable, they are not necessary properties for a short-read alignment program and indicate a slightly different specification than what we, as testers, assumed. The algorithms expect the paired reads to be in a specific direction to map properly and by reverse complementing them, this structure is lost. Additionally, the extension of reads by 20bp before remapping, could extend reads with mismatches and errors, resulting in favoring alternative mapping locations.

We then investigated the properties that systematically fail during BWA testing but not for Bowtie and Bowtie2. Downstream analysis of the BAM files, revealed that the differences in mapping occur mostly for reads that are assigned to the lowest mapping quality or are not uniquely mapped in the genome. By further examining the implementation of BWA we found that in the case of non-uniquely mapped reads, the algorithm is set to report one alignment randomly chosen for each read. This selection is not entirely random, as we found out, since for the same FASTQ file a repeated execution would always produce the same results. The seed for the random number generator is fixed so the random numbers chosen are always the same. This means that in the case where the reads in the input sequence files are at a different position in the file - as it happens after random reshuffling of the reads (MR1), after addition or removal of reads (MR3 and MR4) or after selecting only the mapped reads (MR7) - the reported alignment for each non-uniquely mapped read will be different. Additionally, we found that suppressing the reads that can be aligned to multiple locations did not solve the problem as these reads can be difficult to detect in the BAM file, an issue that we address specifically in the next section. We should note that for low coverage sequencing, this issue might not be always detected as we found out by our simulation runs.

The reason that Bowtie and Bowtie2 do not suffer from this problem is because the pseudo-random number generator is re-initialised for every read, and the seed used to initialise it is a function of the read name, nucleotide string, quality string, and the value specified with a specific parameter "-seed". Therefore the randomly chosen alignments for reporting will always be the same for every read pair irrespectively of where it is located in the input file. On the other hand, since this implementation uses the quality score, this explains why MR8 (Quality score increase of reads) fails for Bowtie and Bowtie2 but not for BWA. Again, the non-uniquely mapped reads reported differ, hence the resulting mapping will be different. We should additionally note that the Bowtie and Bowtie2 implementation uses backtrack to find the alignment, from left to right, and if there are equally good bases, the choice will be random. So when the number of bases are changed, this selection will be changed too, which is another reason why MR5 (Extension of reads) always fails.

Finally, MR9 (Correction of errors or mismatches in the mapped reads) is the only property that gave different results between Bowtie and Bowtie2. We found this property is largely dependent on the input data. When simulated data are used, then there is no fault detected. This is expected, since the error structure in the simulated data is pre-determined and correction of the mismatches in the mapped reads can only improve the mapping. On the other hand, real data can suffer from many sources of bias and a correction of a mismatch can result in mapping of a read to an entirely different location of the genome. Additionally, the implementation first checks the number of mismatches to determine the best alignment taking into account the sum of quality score. When these mismatches are fixed, there might be additional alignments that are of equal equality and the number of alignments can change.

### Effect of MRs in downstream analysis

**Figure 2:**
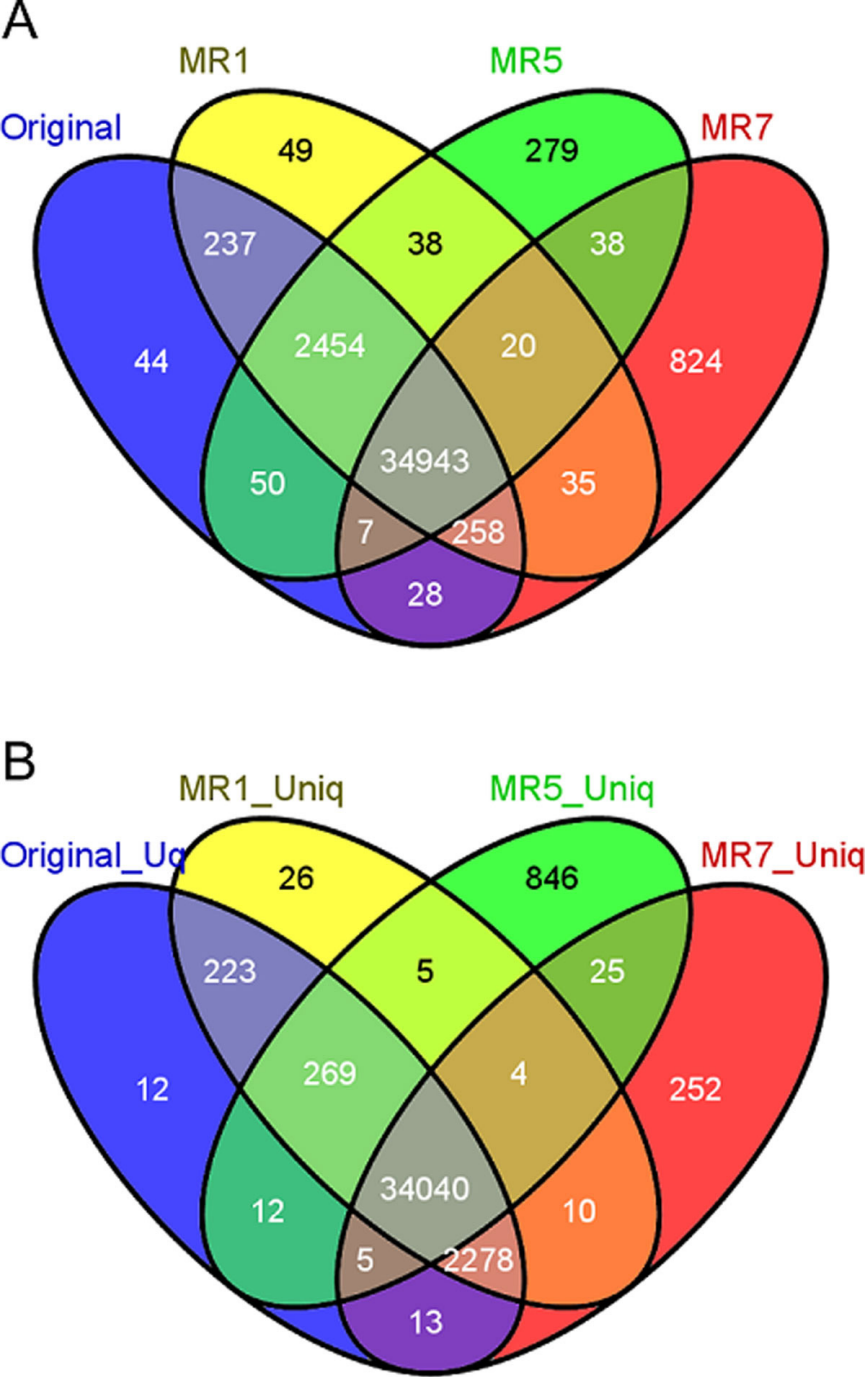
**Number of variants called using original read mapping and mapping after the application of MR1, MR5 and MR7**. A. Using all the reads. B. After removal of non-uniquely mapped reads.

In order to investigate the effect of these properties in downstream WGS or WES analysis, we ran a commonly used pipeline that involves BWA alignment followed by using Genome Analysis Toolkit (GATK) for variant calling [38]. We ran this pipeline for the exome sequenced sample NA12872. Since our MRs do not apply any filtering on the BAM (mapping) files, the analysis was repeated after considering only the uniquely mapped reads. We found that prior to any filtering, the number of variants called is different when we use the Original BAM file, and the resulting BAM files after MR1, MR5 or MR7 (Figure 2A).

We subsequently filtered non-uniquely mapped reads and repeated the variant calling step. We found that there is still discordance between the numbers of variant calls made (Figure 2B). These reads were filtered by mapping quality of 0 as well as reads with an indicated tag of aligning to more than one position ("XT:A:R" and "XT:A:M" for paired-end reads indicating one of the reads is not uniquely mapped). We found that higher quality threshold is needed to achieve concordance between the results and the specified tags are not sufficient to capture all of the non-uniquely mapped reads (given also that the reported ones will vary between the different MRs as we described in the previous section).

### Implication on genomic medicine

Multiple studies have assessed the performance of these and other mapping algorithms [33,39-41] but we were able to do this without the need of an oracle or the direct comparison of the methods. Instead of requiring a mechanism to verify whether an individual test output is correct, the MT technique verifies whether a pair of test outputs conform to a set of domain specific properties, and thus greatly increases the number and variety of test cases that can be applied. Using MT, we detected limitations of BWA, Bowtie and Bowtie2 by demonstrating violations of several user expectations, encoded in the form of metamorphic relations. Although some of these relations are not necessary properties of short-read alignment software, they are important for software validation and software analysis. We detected violations in some MRs that could be characterised as necessary and thus revealing limitations in the implementation of these tools, despite being quite minor. The problems associated with these minor limitations in sequence alignment may be overcome by applying strict quality control filtering in variant calling. Nonetheless, we do believe that it is important to test whether such widely used programs have limitations when we analyse a range of sequencing datasets, as it has also been shown in previous studies [42]. Since one of the major advantages of MT is that it can greatly increase the number of test cases, we can imagine it is possible to extend this framework to automatically and systematically generate many artificial "positive controls" and "negative controls" that can be embedded within each FASTQ file, and be checked for correctness.

## Conclusions

This work illustrates the importance of testing to verify and validate bioinformatics software. Systematic testing can reveal program faults and outcomes that are unexpected and undetected by the user. Such errors or limitations can have tremendous effects in bioinformatics and in scientific computing in general, affecting downstream research or clinical decisions. We proposed a Metamorphic Testing framework for bioinformatics software testing, and demonstrate its utility by testing three widely used sequence aligners.

## Availability of supporting data

The source code and documentation of the MT tool are available at https://sourceforge.net/projects/mr-test/

## Competing interests

The authors declare that they have no competing interests.

## Authors' contributions

JWKH conceived this study. EG and JWKH designed the study and wrote the paper. SHP implemented the software. EG and SHP carried out the analysis. DTH contributed critical software for this project.

## Declarations

The publication costs of this article were funded by the Victor Chang Cardiac Research Institute.

This article has been published as part of *BMC Bioinformatics *Volume 15 Supplement 16, 2014: Thirteenth International Conference on Bioinformatics (InCoB2014): Bioinformatics. The full contents of the supplement are available online at http://www.biomedcentral.com/bmcbioinformatics/supplements/15/S16.
